# Impaired flexibility in patients with tropical spastic paraparesis/HTLV-associated myelopathy: evaluation via pendulum fleximeter

**DOI:** 10.1055/s-0043-1764417

**Published:** 2023-04-14

**Authors:** Caroline Landim, Cristiane Maria Carvalho Costa Dias, Celso Nascimento, Ana Lucia Barbosa Goes, Thessika Hialla Almeida Araújo, Adriele Ribeiro, Francisco Tiago de Oliveira, Humberto Castro-Lima, Ney Boa-Sorte, Bernardo Galvão-Castro

**Affiliations:** 1 Escola Bahiana de Medicina e Saúde Pública, Salvador BA, Brazil.; 2 Universidade Federal da Bahia, Salvador BA, Brazil.; 3 Fundação Oswaldo Cruz, Instituto Gonçalo Moniz, Laboratório Avançado de Saúde Pública, Salvador BA, Brazil.

**Keywords:** Human T-lymphotropic virus 1, Tropical Spastic Paraparesis, Pliability, Articular Range of Motion, Vírus Linfotrópico T Tipo 1 Humano, Paraparesia Espástica Tropical, Maleabilidade, Amplitude de Movimento Articular

## Abstract

**Background** Flexibility is crucial to the harmonious execution of joint movements. While skeletal muscle dysfunction in patients with HTLV-1 can interfere with mobility, it is unclear whether these patients experience reduced flexibility.

**Objective** To evaluate the differences in flexibility between HTLV-1-infected individuals with and without myelopathy compared with uninfected controls. We also investigated whether age, sex, body mass index (BMI), physical activity level, or lower back pain influence flexibility in HTLV-1-infected individuals.

**Methods** The sample consisted of 56 adults, of which 15 did not have HTLV-1, 15 had HTLV-1 without myelopathy, and 26 had TSP/HAM. Their flexibility was assessed using the sit-and-reach test and a pendulum fleximeter.

**Results** No differences in flexibility were observed between the groups with and without myelopathy and controls without HTLV-1 infection using the sit-and-reach test. The pendulum fleximeter results of individuals with TSP/HAM presented the lowest flexibility among the groups with respect to trunk flexion, hip flexion and extension, knee flexion, and ankle dorsiflexion, even after adjusting for age, sex, BMI, level of physical activity, and lower back pain using multiple linear regression models. Additionally, HTLV-1-infected individuals without myelopathy demonstrated reduced flexibility in movements: knee flexion, dorsiflexion, and ankle plantar flexion.

**Conclusions** Individuals with TSP/HAM demonstrated reduced flexibility in most of the movements evaluated by the pendulum fleximeter. Additionally, HTLV-1-infected individuals without myelopathy demonstrated reduced knee and ankle flexibility, potentially representing a marker of myelopathic development.

## Resumo

**Antecedentes** A flexibilidade é fundamental para a execução harmoniosa dos movimentos articulares. Embora a disfunção do músculo esquelético em pacientes com HTLV-1 possa interferir na mobilidade, não está claro se esses pacientes apresentam flexibilidade reduzida.

**Objetivo** Avaliar as diferenças de flexibilidade entre os indivíduos infectados com e sem mielopatia e o grupo controle sem infecção HTLV-1. Também investigamos se idade, sexo, índice de massa corporal (IMC), nível de atividade física ou dor lombar influenciam a flexibilidade em indivíduos infectados pelo HTLV-1.

**Métodos** A amostra foi composta por 56 adultos, dos quais 15 não possuíam HTLV-1, 15 possuíam HTLV-1 sem mielopatia e 26 possuíam TSP/HAM. A flexibilidade foi avaliada por meio do teste de sentar e alcançar e do flexímetro de pêndulo.

**Resultados** Não foram observadas diferenças na flexibilidade entre os grupos com e sem mielopatia no teste de sentar e alcançar. Os resultados do flexímetro pendular dos indivíduos com TSP/HAM apresentaram a menor flexibilidade entre os grupos em relação à flexão do tronco, flexão e extensão do quadril, flexão do joelho e dorsiflexão do tornozelo, mesmo após ajuste para idade, sexo, IMC, nível de atividade física e dor lombar usando modelos de regressão múltipla linear. Além disso, os indivíduos infectados pelo HTLV-1 sem mielopatia demonstraram redução da flexibilidade nos movimentos de flexão do joelho, dorsiflexão e flexão plantar do tornozelo.

**Conclusão** Indivíduos com TSP/HAM demonstraram redução da flexibilidade na maioria dos movimentos avaliados pelo flexímetro pendular. Além disso, indivíduos infectados pelo HTLV-1 sem mielopatia demonstraram redução da flexibilidade do joelho e tornozelo, representando potencialmente um marcador de desenvolvimento mielopático.

## INTRODUCTION

The adult T cell leukemia virus-1 (HTLV-1), a delta retrovirus also known as Human T-lymphotropic virus type 1, was the first retrovirus to be isolated in humans. 1 The highest HTLV-1 prevalence is found in Japan, Africa, the Caribbean, Melanesia, Iran, and South America. 2 It is estimated that at least 5 to 10 million people harbor this virus worldwide. 2 Brazil, which potentially harbors 800,000 people with HTLV-1, represents the country with the largest number of carriers on the American continent. Transmission occurs through sexual relations, transfusion/transplantation of blood/organs infected with the virus, and from mother-to-child, mainly by breastfeeding. 2 The state of Bahia, located in northeastern Brazil, is one of the most affected by HTLV-1, with ∼130,000 infected persons. 3 Salvador is the state capital with highest prevalence in Brazil, with an estimated 50,000 infected individuals, corresponding to approximately 2% of this city's population. 4 5

The HTLV-1 is etiologically linked to adult T cell leukemia-lymphoma (ATLL), tropical spastic paraparesis/HTLV-1-associated myelopathy (TSP/HAM), uveitis and infective dermatitis. 6 7 8 9 10 Due to systemic involvement, many other diseases have been associated with HTLV-1 infection, such as polymyositis, sinusitis, broncho-alveolar pneumonia, keratoconjunctivitis sicca, bronchiectasis, encephalopathy, and neurogenic bladder. 2 11 12 13

Manifestations associated with TSP/HAM, a chronic, progressive demyelinating disease that mainly affects the thoracic region of the spinal cord, generally appear after 40 years of age. 14 Additionally, white matter lesions have also been observed. 15 The estimated lifetime risk of an asymptomatic carrier to develop TSP/HAM ranges from 0.25 to 3.7%. 15 This disease is characterized by spastic paraparesis, muscle weakness, and stiffening in the lower limbs, as well as disorders of the bladder and bowel sphincter and chronic pain, especially in the lower back and lower limbs. 13 These alterations can lead to gait impairment, reduced functional movement and postural changes. 16 Consequently, TSP/HAM may lead to reduced joint flexibility and can interfere in the harmonic execution of lower limb and trunk movements, contributing to a reduction in functional capacity and risk of fall. 17 18 19

A previous study provided preliminary evidence indicating that patients with TSP/HAM present reduced joint flexibility and, consequently, mobility, resulting in decreased functional capacity compared with uninfected individuals. 20

Joint flexibility is the range of motion (ROM) in a joint or group of joints, or the ability of joints to move effectively through a complete range of motion. 21 Adequate range of motion requires synergy between joint mobility and the elasticity of surrounding soft tissue (muscle, cartilage, ligaments, and tendons). 22 23 The sit and reach test, whose values are expressed in centimeters, is the test most widely used to assess flexibility. 22 Flexibility can also be assessed using a pendulum fleximeter, which records the angles of joint movements, expressed in degrees. 24 25

In addition to endogenous factors (age, sex, biological, body mass index [BMI], and somatotype), exogenous factors (time of day, ambient temperature, exercise, and fatigue) may also influence joint flexibility. 26 A study assessing hip and ankle ROM during the straight leg raise neurodynamic test found that joint motion can be influenced by gender, weight, BMI, and physical activity level. 27

It is well known that changes in joint flexibility can affect mobility and increase the risk of falls. Therefore, it is important to investigate the changes in flexibility in patients with TSP/HAM. The current study aimed to more accurately evaluate differences in flexibility between infected individuals with and without myelopathy, comparing measurements obtained via pendulum fleximeter to a control group without the HTLV-1 infection. Additionally, we investigated whether the confounding variables of age, sex, BMI, level of physical activity, and lower back pain influence flexibility in HTLV-1-infected individuals.

## METHODS

### Study design, location, and population

The present exploratory, analytical, cross-sectional controlled study was conducted between April 2018 and December 2019 in a non-probabilistic sample at the Integrated Multidisciplinary HTLV/Neurosciences Center (CHTLV) at the Bahiana School of Medicine and Public Health (EBMSP). This outpatient clinic provides comprehensive biopsychosocial care to the public, with support provided by the National Unified Health Care System (SUS), including general medical treatment, laboratory diagnosis, psychological counseling, and physical therapy. 28 Since its inauguration in 2002, a total of 2,169 HTLV-infected patients consulted at the CHTLV, approximately less than 50% of whom are evaluated regularly. Of these, 2,145 (98.9%) are cases of HTLV-1 infection, while 24 (1.1%) individuals were diagnosed with HTLV-2. Almost all patients (98%) reside in the city of Salvador (Bahia, Brazil), were aged between 5 and 93 years (mean: 49.8, standard deviation [SD]: 15.9), and 70.3% (1,525) are female. Most (84.6%) self-reported black or brown skin color, 73% had less than 8 years of schooling, and half earned the equivalent of one Brazilian monthly wage (∼US$200).

The patients were recruited sequentially during routine neurological evaluations by a single trained neurologist. All HTLV-1-infected patients, with or without myelopathy, aged between 18 and 65 years were invited to participate, as well as individuals without HTLV infection, who served as controls for comparison purposes. Individuals with TSP/HAM were diagnosed as ‘definite’, according to the criteria established by De Castro-Costa et al. (2006). 29

Briefly, definite TSP/HAM is considered when non-remitting progressive spastic paraparesis sufficiently impairs a patient's gait to the point of being perceptible. The presence of HTLV-1 antibodies in serum and cerebrospinal fluid (CSF) must be confirmed by Western blot and/or C-reactive protein (CRP) positivity for HTLV-1 in blood and/or CSF. While sensory symptoms or signs may or may not be present, when present they remain subtle, lacking a clear-cut level of sensory perception. Urinary and anal sphincter signs or symptoms may or may not be present. Any other disorders that resemble TSP/HAM should be excluded through appropriate laboratory and clinical evaluations. To calculate the sample size, we used the data of Santos et al., 20 who assessed the flexibility of individuals without infection, HTLV-1-positive individuals, and HTLV-1 patients with myelopathy using the “sit and reach” test. Asymptomatic and uninfected HTLV-1 groups were included based on the difference in mean values.

The following parameters were used: mean (SD) of flexibility between uninfected and asymptomatic subjects of 29.2 (7.5) and 23.4 (7.9) cm, respectively, power of 80%, and α-error of 0.05, resulting in a sample of 29 subjects in each group, with an increase of 20% due to possible losses and adjustments for multivariate analysis. Calculations were performed with the OpenEpi program (Centers for Disease Control and Prevention, USA), version 3.

All included patients signed a term of informed consent. This study was approved the Institutional Review Board of EBMSP (protocol number 2.480.700/2018).

Exclusion criteria consisted of any other causes of motor deficiency (e.g., stroke, trauma), wheelchair use, evidence of coinfection, e.g., syphilis, hepatitis B and C, human immunodeficiency virus (HIV), or any other diseases that could lead to neurological impairment (e.g., vitamin B12 deficiency, diabetes mellitus, multiple sclerosis, neuroschistosomiasis). The HTLV-1-positive individuals who did not meet the criteria for definite TSP/HAM (i.e., possible/probable TSP/HAM) were also excluded.

All HTLV-1 patients without myelopathy were submitted to two neurological scales to confirm the absence of neurological abnormalities: 1) expanded disability status scale (EDSS) 30 and Osame's motor disability score (OMDS). 31 Eligible scores for HTLV-1 patient inclusion were OMDS <1 and EDSS ≤1. According to the World Health Organization (WHO), TSP/HAM is clinically defined by EDSS ≥2 and/or OMDS ≥1. 32

### Evaluations

A semi-structured questionnaire was applied to collect information on the following variables: sex, age, weight, height, BMI, marital status, self-reported skin color, education, family income (recorded in multiples of minimum monthly wage). The BMI was categorized as obese (BMI ≥ 25 kg/m2) or non-obese (BMI < 25 kg/m2). The brief pain inventory (BPI), validated in Portuguese (Brazilian), was applied to assess the frequency and location of pain experienced by individuals over the previous 24 hours. 33 34 Physical activity level was evaluated through the international physical activity questionnaire (IPAQ), which estimates weekly time spent on physical activities and classifies activity levels as active (≥150 minutes/week) or inactive (<150 minutes/week). 35

To measure each participant's flexibility, the sit and reach test was employed to measure, in centimeters, the degree of elongation of the posterior part of the trunk and lower limbs. 36 A pendulum fleximeter (Sanny, American Medical SA, São Bernardo do Campo, SP, Brazil) was used to evaluate flexion, measuring angles in degrees through active movements, considering: the extension of the trunk, hips and knees, adduction and abduction, internal and external rotation of the left and right hips, as well as dorsiflexion and plantar flexion in the left and right ankles. 37

### Statistical analysis

Quantitative variables were assessed for normal distribution using the Shapiro-Wilk test. Results were expressed as means ( ±  SD) when normal distributions were verified, while medians (with 25–75 ^th^ percentiles) were used for non-normal distributions. Absolute and relative frequencies were described for categorical variables. Comparisons among sociodemographic and clinical characteristics were performed using the χ2 test or the Fisher exact test where indicated (categorical variables). Analysis of variance (ANOVA) was performed using the Bonferroni post hoc or the non-parametric Kruskal-Wallis tests, with the Dunn-Bonferroni post-test correction where indicated (continuous variables).

Individual sit and reach test values, as well as group medians of the HTLV infection status, were plotted via dot-plot graphs. Multivariate linear regression analysis was employed on datasets containing the angles measured using a pendulum fleximeter, while adjusting for confounding factors (sex, age, BMI, physical activity, and lower back pain), considering the HTLV-1 ^(−)^ group as a reference. Pseudo r2 values and β coefficients with respective 95% confidence intervals (95% CI) are shown in each adjusted model. The *p* -values < 0.05 were considered statistically significant. All statistical analyses were performed using the Stata (Stata Corp LLC., Texas, TX, USA) software, version 13.0, for Mac, and graphs were constructed using Prism (GraphPad Software, San Diego, California USA), version 9.1.2, for Mac.

## RESULTS

**Figure 1:**
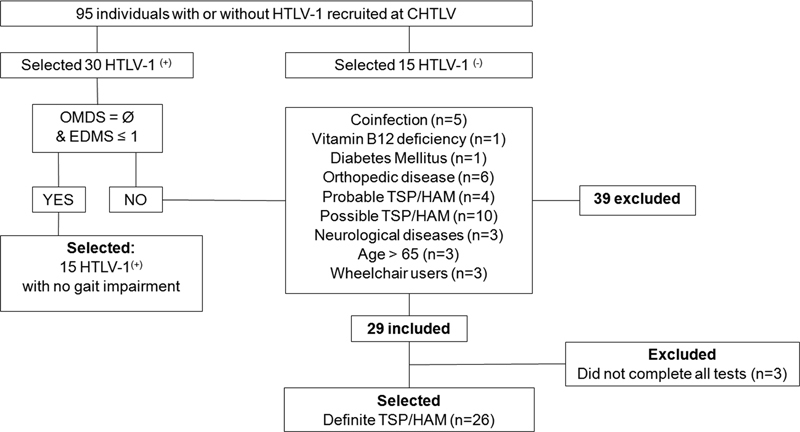
Flowchart detailing participant selection and inclusion/exclusion criteria. **Abbreviations:** HTLV-1, human T cell lymphotropic virus type 1; TSP/HAM, tropical spastic paraparesis/HTLV-1-associated myelopathy.

A total of 95 individuals were recruited, 80 of whom were HTLV-1 positive. In all, 39 HTLV-1-positive individuals were excluded due to non-eligibility. The final sample consisted of 26 HTLV-1 positive individuals with definite TSP/HAM (TSP/HAM), 15 HTLV-1-positive individuals without myelopathy (HTLV-1 ^(+)^ ), and 15 individuals without infection (HTLV-1 ^(−)^ ) ( Figure 1 ).

Sociodemographic and anthropometric data are shown in Table 1 . A homogeneous distribution was observed between the three study groups with respect to the variables of sex, self-reported skin color, education, BMI, physical activity, and low back pain. However, significant differences were observed in the categories of age, employment status, and family income. Participants in the definite TSP/HAM group were older and presented lower median BMI levels than the HTLV-1 ^(+)^ and HTLV-1 ^(−)^ groups ( Table 1 ). The HTLV-1 ^(+)^ group was composed of younger individuals, with a median age of 39 (range: 30–52) years; all individuals reported income below the minimum wage, and 73% were considered overweight. The HTLV-1 ^(−)^ group reported significantly higher family income than the HTLV-1 ^(+)^ and TSP/HAM groups.

**Table 1:** Sociodemographic and clinical characteristics of 56 individuals with and without HTLV-1 and with definite HAM/TSP

				Total N (%)	HTLV-1 ^(−)^ N (%)	HTLV-1 ^(+)^ N (%)	TSP/HAM N (%)	*p* -value
**Age (years), median-IQR**				50.5 (46.5–55)	53 (49–54)	39 (30–52)	51.5 (48–58)	0.012**
**Range**				28–65	47–58	24–58	28–65	
** BMI (Kg/m ^2^ ) **				26.9 (22.7–29.4)	27.5 (25.5–28.7)	28.2 (24.2–31.2)	24.3 (20.9–28.7)	0.197**
**Range**				17.7–38.6	21.4–36	19.9–34.5	17.7–38.6	
** BMI ≥ 25 kg/m ^2^**	No	42 (75)	3 (20)	4 (26.7)	14 (53.8)	0.074*
	Yes		14 (25)	12 (80)	11 (73.3)		12 (46.1)
**Sex**	Female	42 (75)	11 (73.3)	13 (86.7)	18 (69.2)	0.528*
	Male		14 (25)	4 (26.7)	2 (13.3)		8 (30.7)
**Skin color**	Black	24 (42.8)	3 (20)	7 (46.7)	14 (53.9)	0.121*
	Other		32 (57.2)	12 (80)	8 (53.3)		12 (46.1)
**Education (years)**	≤ 8	34 (60.7)	12 (80)	12 (80.0)	10 (38.5)	0.487*
	8 - 11		9 (16)	3 (20)	1 (6.7)		4 (15.3)
	≥ 11		13 (23.3)	−	2 (10.5)		12 (46.1)
**Employment status**	Employed	34 (60.7)	12 (80)	12 (80.0)	10 (38.5)	0.002*
	Unemployed			9 (16)	3 (20)		1 (6.7)	4 (15.3)
	Retired			13 (23.3)	−		2 (10.5)	12 (46.1)
**Family Income (MW)**	≤1	44 (78.6)	8 (53.3)	15 (100)	21 (80.8)	0.007*
	>1	12 (21.4)	7 (46.7)	−	5 (19.2)	
**IPAQ classification**	Active	14 (25)	4 (26.7)	1 (6.7)	9 (34.6)	0.152*
	Inactive		42 (75)	11 (73.3)	14 (93.3)		17 (65.4)
**Lower back pain**	No	13 (23.21)	3 (20)	4 (26.67)	6 (23.08)	> 0.999*
	Yes		43 (76.79)	12 (80)	11 (73.33)		20 (76.92)

**Abbreviations:** HTLV-1, human T cell lymphotropic virus type 1; TSP/HAM, tropical spastic paraparesis/ HTLV-1-associated myelopathy; BMI, body mass index; Ipaq, international physical activity questionnaire. **Notes:** *Fisher exact test; **Kruskal-Wallis test.

**Figure 2:**
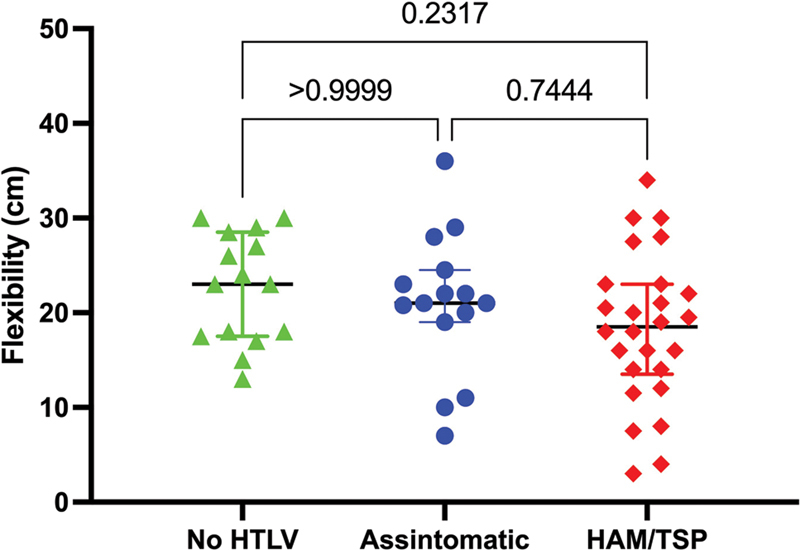
Flexibility performance on the sit and reach test comparing HTLV-1 ^(-)^ , HTLV-1 ^(+)^ and definite TSP/HAM groups. Dot plots representative of individual measurements. The Kuskal-Wallis test was used.

When analyzing flexibility using the sit and reach test, no statistically significant differences were observed between the three groups ( *p* = 0.232). However, the data shown in Figure 2 revealed a gradual reduction in median (25–75 ^th^ percentile) values across the three groups: the median value obtained for the HTLV-1 ^(−)^ group was 23.0 (17.5–28.5) cm, higher than the HTLV-1 ^(+)^ group (median: 21 cm, range: 19–24.5 cm) and the definite TSP/HAM group (median: 18.5 cm, range: 14–23) cm). Most participants (76.8%) reported pain within the last 24 hours. The most frequent site of pain in all groups was the lumbar region (69.64%): 9/15 (60.0%) in the HTLV-1 ^(−)^ group, 8/15 (53.33%) in the HTLV-1 ^(+)^ group, and 22/26 (84.62%) in the HAM/TSP group.

Table 2 describes flexibility evaluations, measured in angular degrees using a pendulum fleximeter, of the trunk, hip, knee, and ankle, as well as univariate analysis of group median values. No significant associations were observed between the pain site and flexibility (data not shown). With regard to trunk extension, the TSP/HAM group presented significantly lower values than the HTLV-1 ^(+)^ and HTLV-1 ^(−)^ groups. Regarding right and left hip flexion and right and left hip extension, the definite TSP/HAM group presented less flexibility compared with the HTLV-1 ^(+)^ and HTLV-1 ^(−)^ groups.

**Table 2:** Flexibility comparisons (measured in degrees) between groups of individuals with and without HTLV-1 and those with definite TSP/HAM

Flexibility (degrees)		HTLV-1 ^(−)^	HTLV-1 ^(+)^	TSP/HAM	*p* -value
**Trunk**	Flexion	80 (65–90)	70 (50–90)	25 (10–54)	a ^3^ p < 0.001; b ^2^ p < 0.01
	Extension	20 (18–25)	25 (15–30)	20 (15–30)	a ^3^ p < 0.001; b ^1^ p < 0.05
**Right hip**	Flexion	60 (45–77)	55 (35–70)	30 (20–45)	a ^3^ ; b ^3^ p < 0.001
	Extension	31 (20–62)	33(20–45)	10 (3–20)	a ^2^ p < 0.01; b ^3^ p < 0.001
	Abduction	55 (45–65)	90 (60–120)	39 (10–60)	b ^3^ p < 0.001; c ^1^ p < 0.05
	Adduction	18 (15–26)	18 (10–39)	18 (15–28)	−
	Internal rotation	26 (15–41)	20 (15–40)	20 (10–35)	−
	External rotation	45 (35–46)	40 (20–45)	25 (11–35)	b ^1^ p < 0.05
**Left hip**	Flexion	59 (45–79)	50 (30–60)	28 (16–40)	a ^2^ p < 0.01; b ^3^ p < 0.001
	Extension	40 (25–55)	40 (25–50)	10 (5–20)	a ^3^ ; b ^3^ p < 0.001
	Abduction	60 (53–75)	70 (55–110)	31 (10–55)	a ^3^ p < 0.001; b ^2^ p < 0.01
	Adduction	15 (9–32)	20 (15–30)	19 (14–35)	−
	Internal rotation	25 (20–30)	30 (10–48)	24 (15–45)	−
	External rotation	31 (26–35)	22 (15–35)	19 (5–30)	b ^2^ p < 0.01
**Right knee**	Flexion	105 (90–120)	100 (90–105)	62.5 (30–100)	b ^2^ p < 0.01
	Extension	18 (15–26)	20 (12–30)	10 (6–20)	b ^2^ p < 0.01
**Left knee**	Flexion	108 (95–125)	110 (90–115)	40 (12–95)	a ^3^ p < 0.001; b ^1^ p < 0.05
	Extension	25 (15–34)	15 (10–35)	11 (8–20)	b ^2^ p < 0.01
**Right ankle**	Dorsiflexion	25 (10–30)	15 (10–25)	11.5 (5–20)	b ^2^ p < 0.01
	Plantar flexion	25 (15–35)	10 (8–20)	15 (5–25)	b ^2^ c ^2^ p < 0.01
**Left ankle**	Dorsiflexion	15 (11–25)	20 (10–30)	10.5 (5–15)	b ^1^ p < 0.05
	Plantar flexion	17 (8–27)	12 (8–15)	10 (5–20)	−

**Abbreviations:** HTLV-1, human T cell lymphotropic virus type 1; TSP/HAM, tropical spastic paraparesis / HTLV-1-associated myelopathy. **Notes:** Letters indicate comparisons between groups: **a** : comparison between definite HAM/TSP versus HTLV-1 ^(+)^ ; **b** : comparison between definite HAM/TSP versus HTLV-1 ^(−)^ ; **c** : comparison between HTLV-1 ^(+)^ versus HTLV-1 ^(−)^ . Numbers indicate level of significance: ^1^ *p* -value < 0.05; ^2^ *p* -value < 0.01; ^3^ *p* -value < 0.001. All comparisons were performed using the Kruskal-Wallis test with Dunn-Bonferroni post-test correction. Values are represented as medians and interquartile range.

Multiple linear regression was applied to analyze flexibility, measured in degrees using a pendulum fleximeter. Four models were employed to adjust for the confounding variables of age, sex, BMI, level of physical activity, and lower back pain in the analyzed groups ( Tables 3 , 4 , and 5 ). In the analyzed models, after adjusting for confounders, individuals with TSP/HAM presented consistently lower trunk flexion values (measured in degrees) across all models ( *p* < 0.001). Surprisingly, HTLV-1 ^(+)^ individuals presented higher median trunk extension values than HTLV-1 ^(−)^ individuals in all models ( *p* < 0.01) ( Table 3 ).

**Table 3:** Mean variation (β coefficient) analysis of trunk flexion and extension with respective 95% CI values in the HTLV-1 ^(+)^ and TSP/HAM groups, adjusted for age, BMI, physical activity level and lower back pain

Flexibility (degrees)	Model 1	*p* -value	Model 2	*p* -value	Model 3	*p* -value	Model 4	*p* -value
	Beta (95% CI)		Beta (95% CI)		Beta (95% CI)		Beta (95% CI)	
**Trunk Flexion**								
adjusted r ^2^	0.2299		0.2233		0.2073		0.1868	
**Reference**	**HTLV-1** ^(−)^		**HTLV-1** ^(−)^		**HTLV-1** ^(−)^		**HTLV-1** ^(−)^	
HTLV-1 ^(+)^	-14.99	0.11	-16.05	0.09	-16.01	0.10	-12.99	0.15
	(-33.75/3.76)		(-35.27/3.15)		(-35.45/3.44)		(-31/5.01)	
TSP/HAM	-32.08	**<0.001**	-33.87	**<0.001**	-33.91	**<0.001**	-31.91	**<0.001**
	(-47.07/-17.09)		(-49.23/-18.51)		(-49.46/-18.36)		(-48.04/-15.78)	
**Trunk extension**								
adjusted r ^2^	0.1871		0.2143		0.215		0.2436	
HTLV-1 ^(+)^	9.66	**0.01**	9.44	**0.01**	9.68	**0.01**	11	**0.002**
	(2.09/ 17.22)		(1.86/17.03)		(2.09/17.28)		(4.06/17.94)	
TSP/HAM	0.39	0.89	-0.56	0.25	-0.74	0.80	0.87	0.77
	(-5.64/6.43)		(-6.62/5.49)		(-6.82/5.32)		(-5.33/7.09)	

**Abbreviations:** 95% CI, 95% confidence interval; HTLV-1, human T cell lymphotropic virus type 1; TSP/HAM, tropical spastic paraparesis / HTLV-1 associated myelopathy; IPAQ, international physical activity questionnaire. **Notes:** Model 1: adjusted for age; Model 2: adjusted for age, sex, and body mass index; Model 3: adjusted for age, sex, BMI, and physical activity level; Model 4: adjusted for age, sex, BMI, physical activity level, and lower back pain. All comparisons were performed using the Kruskal-Wallis test and Dunn-Bonferroni post-test correction.

A separate comparison of trunk flexion between the TSP/HAM group and HTLV-1 ^(+)^ individuals revealed lower values (-17.63; 95% CI: -35.75/0.48; *p* = 0.056) with borderline significance in the former group, which were not significant after adjusting for confounders (-18.10; 95% CI: -37.43/1.23; *p* = 0.066). Regarding trunk extension, TSP/HAM individuals presented significantly lower median values compared with the HTLV-1 ^(+)^ group across all models (data not shown).

Regarding flexibility in hip movements, comparing the HTLV-1 ^(+)^ and TSP/HAM groups to HTLV-1 ^(−)^ individuals, statistical significance was consistently observed across the adjusted models for right and left hip flexion, right and left hip extension, as well as right hip external rotation in the TSP/HAM group. With regard to left hip extension, significance was observed in the HTLV-1 ^(+)^ group across all four multivariate models. Furthermore, considering left hip abduction, significantly lower results were found in TSP/HAM group only in Model 1 ( *p* = 0.04). Moreover, significance was also observed across Models 2 to 4 in the TSP/HAM group when left hip external rotation values were analyzed ( Table 4 ).

**Table 4:** Mean variation (β coefficient) analysis of hip flexibility with respective 95% CI values in the HTLV-1 ^(+)^ and TSP/HAM groups, adjusted for age, sex, BMI, physical activity level, and lower back pain

Flexibility (degrees)	Model 1	*p* -value	Model 2	*p* -value	Model 3	*p* -value	Model 4	*p* -value
	Beta (95% CI)		Beta (95% CI)		Beta (95% CI)		Beta (95% CI)	
**Right hip flexion**								
adjusted r ^2^	0.2387		0.2086		0.2583		0.2253	
**Reference**	** HTLV-1 ^(−)^**		** HTLV-1 ^(−)^**		** HTLV-1 ^(−)^**		** HTLV-1 ^(−)^**	
HTLV-1 ^(+)^	-8.2	0.3	-10.18	0.25	-9.12	0.36	-7.35	0.37
	(-24.13/7.71)		(-27.93/7.56)		(-26.4/8.16)		(-23.57/8.86)	
TSP/HAM	-28.69	**<0.001**	-28.06	**<0.001**	-28.89	**<0.001**	-27.85	**<0.001**
	(-42.32/-15.05)		(- 42.25/-13.88)		(-42.68/-15.05)		(-42.37/-13.3)	
**Left hip flexion**								
adjusted r ^2^	0.1586	0.12	0.1604		0.1751	0.19	0.1722	
HTLV-1 ^(+)^	-14.87		-13.43	0.16	-12.61		-8.74	0.32
	(-33.75/4.01)		(-32.66/6.80)		(-31.72/6.49)		(-26.36/8.87)	
TSP/HAM	-26.53	**<0.001**	-27.14	**<0.001**	-27.75	**<0.001**	-26.35	**<0.001**
	(-41.61/-11.44)		(-42.51/-11.76)		(-43.02/-12.48)		(-42.13/-10.58)	
**Right hip extension**								
adjusted r ^2^	0.2092		0.1959		0.1908		0.1842	
HTLV-1 ^(+)^	-1.04	0.89	-0.67	0.93	-1.69	0.84	-1.28	0.7
	(-17.38/15.3)		(-17.28/15.94)		(-18.55/17.17)		(-18.26/15.68)	
TSP/HAM	-21.66	**<0.001**	-21.75	**<0.001**	-22.8	**<0.001**	-23.1	**<0.001**
	(-34.72/-8.6)		(-34.94/-8.56)		(-36.28/-9.32)		(-36.67/-9.54)	
**Left hip extension**								
adjusted r ^2^	0.2434		0.2315		0.2207		0.1892	
HTLV-1 ^(+)^	-1.61	0.85	-2.15	0.8	-2.45	0.78	3.72	0.64
	(-18.56/15.35)		(-19.58/15.27)		(-20.05/15.14)		(-12.5/19.95)	
TSP/HAM	-25.11	**<0.001**	-26.46	**<0.001**	-26.24	**<0.001**	-19.28	**0.01**
	(-38.67/-11.56)		(-40.4/-12.53)		(-40.3/-12.17)		(-33.82/-4.74)	
**Right hip abduction**								
adjusted r ^2^	0.3029		0.3344		0.3267		0.266	
HTLV-1 ^(+)^	30.18	**0.02**	32.99	**0.01**	32.45	**0.01**	27.05	**0.03**
	(4.34/56.02)		(7.24/58.74)		(6.5/58.41)		(2.05/52.05)	
TSP/HAM	-15.58	0.13	-11.45	0.26	-11.05	0.28	-7.27	0.51
	(-36.23/5.06)		(-32.04/9.12)		(-31.79/9.68)		(-29.67/15.11)	
**Left hip abduction**								
adjusted r ^2^	0.1614		0.1934		0.1826		0.1632	
HTLV-1 ^(+)^	12.49	0.39	14.92	0.3	15.45	0.29	8.99	0.51
	(-16.47/41.46)		(-14.04/43.89)		(-13.77/44.69)		(-18.23/36.22)	
TSP/HAM	-23.42	**0.04**	-19.08	0.1	-19.48	0.10	-16.27	0.18
	(-46.57/-0.27)		(-42.24/4.06)		(-42.84/3.87)		(-40.66/8.11)	
**Right hip aduction**								
adjusted r ^2^	-0.0055		-0.0229		-0.0244		-0.0239	
HTLV-1 ^(+)^	8.33	0.21	8.61	0.2	9.58	0.16	9.15	0.18
	(-4.93/21.61)		(-4.88/22.11)		(-4.07/23.24)		(-4.53/22.85)	
TSP/HAM	-0.95	0.85	-1.01	0.84	-0.02	0.99	-0.29	0.95
	(-11.56/9.65)		(-11.73/9.69)		(-10.95/10.89)		(-10.65/11.23)	
**Right hip aduction**								
adjusted r ^2^	0.011		0.0739		0.0817		0.0481	
HTLV-1 ^(+)^	5.18	0.5	3.58	0.64	3.01	0.69	0.38	0.95
	(-10.35/20.71)		(-11.74/18.91)		(-12.28/18.31)		(-13.66/14.43)	
TSP/HAM	7.42	0.23	3.73	0.44	5.15	0.4	5.08	0.42
	(-4.98/19.84)		(-7.52/16.98)		(-7.07/17.38)		(-7.49/17.66)	
**Right hip internal rotation**								
adjusted r ^2^	0.0551		0.47		0.0277		0.0577	
HTLV-1 ^(+)^	3.44	0.58	2.87	0.65	2.91	0.65	6.49	0.28
	(-8.99/15.88)		(-9.86/15.61)		(-9.98/15.81)		(-5.66/18.65)	
TSP/HAM	-4.61	0.35	-5.77	0.26	-5.81	0.26	-6.69	0.22
	(-14.55/5.32)		(-15.95/4.4)		(-16.11/4.5)		(-17.58/4.19)	
**Left hip internal rotation**								
adjusted r ^2^	-0.0203		-0.0233		-0.0256		-0.0643	
HTLV-1 ^(+)^	7.34	0.35	6.25	0.43	6.72	0.4	4.86	0.51
	(-8.43/23.12)		(-9.85/22.36)		(-9.44/22.89)		(-10.07/19.81)	
TSP/HAM	2.81	0.65	1.16	0.85	0.8	0.8	2.95	0.65
	(-9.78/15.42)		(-11.71/14.04)		(-12.11/13.73)		(-10.42/16.34)	
**Right hip external rotation**								
adjusted r ^2^	0.214		0.2311		0.2155		0.1506	
HTLV-1 ^(+)^	-5.02	0.36	-5.88	0.29	-5.85	0.3	-6.97	0.20
	(-16.15/6.09)		(-17.1/5.33)		(-17.21/5.5)		(-17.84/3.89)	
TSP/HAM	-16.56	**<0.001**	-18.05	**<0.001**	-18.07	**<0.001**	-18.16	**<0.001**
	(-25.45/-7.67)		(-27.02/-9.08)		(-27.15/-8.99)		(-27.9/-8.43)	
**Left hip external rotation**								
adjusted r ^2^	0.0727		0.0858		0.0763		0.0731	
HTLV-1 ^(+)^	1.21	0.85	1.39	0.83	1.11	0.86	-1.26	0.83
	(-11.81/14.24)		(-11.79/14.59)		(-12.18/14.4)		(-13.3/10.77)	
TSP/HAM	-9.31	0.07	-10.49	**0.05**	-10.28	**0.05**	-12.35	**0.02**
	(-19.73/1.09)		(-21.04/0.5)		(-20.9/0.34)		(-23.13/-1.56)	

**Abbreviations:** 95% CI, 95% confidence interval; HTLV-1, human T cell lymphotropic virus type 1; TSP/HAM, Tropical spastic paraparesis / HTLV-1 associated myelopathy; IPAQ, international physical activity questionnaire. **Notes:** Model 1: adjusted for age; Model 2: adjusted for age, sex, and body mass index; Model 3: adjusted for age, sex, BMI, and physical activity level; Model 4: adjusted for age, sex, BMI, physical activity level, and low back pain. All comparisons were performed using the Kruskal-Wallis test and Dunn-Bonferroni post-hoc corrections.

A subcomparison of differences in hip movement between TSP/HAM and HTLV-1 ^(+)^ groups revealed significantly worse values across all models with regard to the flexion, extension, and abduction movements of both hips, as well as the external rotation of the right hip (data not shown).

Multivariate linear regression analysis of knee and ankle flexibility ( Table 5 ) revealed consistently significantly worse results for the TSP/HAM group in the categories of right knee flexion, right ankle dorsiflexion, and right ankle plantar flexion. However, with regard to the last one, significance was observed in the HTLV-1 ^(+)^ group only in Models 2 and 3 ( Table 5 ).

**Table 5:** Mean variation (β coefficient) analysis of knee and ankle flexibility with respective 95% CI values in the HTLV-1(+) and TSP/HAM groups, adjusted for age, sex, BMI, activity level, and lower back pain

Flexibility (degrees)	Model 1	*p* -value	Model 2	*p* -value	Model 3	*p* -value	Model 4	*p* -value
	Beta (95% CI)		Beta (95% CI)		Beta (95% CI)		Beta (95% CI)	
**Right knee flexion**								
**Reference**	**HTLV-1** ^(−)^		**HTLV-1** ^(−)^		**HTLV-1** ^(−)^		**HTLV-1** ^(−)^	
adjusted r ^2^	0.1532		0.1346		0.1214		0.0997	
HTLV-1 ^(+)^	-10.13	0.47	-9.84	0.49	-9.39	0.52	-12.32	0.36
	(-38.32/17.96)		(-38.8/19.12)		(-38.64/19.86)		(-39.31/14.66)	
TSP/HAM	-36.9	**<0.001**	-35.17	**<0.001**	-35.51	**<0.001**	-36.43	**<0.001**
			(-58.32/-12.02)		(-58.88/-12.13)		(-60.6/-12.25)	
**Right knee extension**								
adjusted r ^2^	-0.0498		-0.0585		-0.0709		0.0274	
HTLV-1 ^(+)^	-0.94	0.93	-3.48	0.76	-3.95	0.73	-2.26	0.53
	(-23.27/21.38)		(-26.35/19.37)		(-27/19.1)		(-10.14/5.61)	
TSP/HAM	-0.27	0.97	-2.19	0.81	-1.84	0.84	-7.48	**0.03**
	(-18.11/17.56)		(-20.47/16.08)		(-20.26/16.57)		(-14.54/-0.42)	
**Left knee extension**								
adjusted r ^2^	0.0381		-0.0422		-0.0611		0.0823	
HTLV-1 ^(+)^	-5.09	0.61	-6.04	0.54	-5.83	0.56	4.65	0.28
	(-24.5/14.31)		(-25.87/13.78)		(-25.88/14.22)		(-4/13.3)	
TSP/HAM	-4.29	0.58	-6.22	0.43	-6.38	0.42	-3.46	0.37
	(-19.8/11.21)		(-22.07/9.62)		(-22.41/9.64)		(-11.21/4.28)	
**Right ankle dorsiflexion**								
adjusted r ^2^	0.0692		0.0458		0.0278		0.0392	
HTLV-1 ^(+)^	-1.65	0.69	-2.34	0.59	-2.42	0.58	-6.75	0.10
	(-10.07/6.76)		(-11.04/6.34)		(-11.22/6.37)		(-14.95/1.4)	
TSP/HAM	-7.61	**0.02**	-8.04	**0.02**	-7.98	**0.02**	-9.31	**0.02**
	(-14.33/-0.87)		(-14.99/-1.09)		(-15.02/-0.95)		(-16.65/-1.97)	
**Left Ankle Dorsiflexion**								
adjusted r ^2^	0.0161		0.0750		0.0569		-0.0314	
HTLV-1 ^(+)^	1.57	0.72	1.14	0.78	1.09	0.81	-5.28	0.14
	(-7.49/10.65)		(-7.82/10.12)		(-7.98/10.17)		(-12.34/1.78)	
TSP/HAM	-5.06	0.16	-4.1	0.25	-4.06	0.26	-3.93	0.21
	(-12.31/2.18)		(-11.27/3.06)		(-11.32/3.19)		(-10.26/2.38)	
**Right Ankle Plantar flexion**								
adjusted r ^2^	0.0755		0.0764		0.0735		0.0179	
HTLV-1 ^(+)^	-7.49	0.06	-8.61	**0.04**	-8.38	**0.04**	-2.54	0.56
	(-15.57/0.57)		(-16.84/-0.38)		(-16.64/-0.12)		(-11.39/6.32)	
TSP/HAM	-8.19	**0.01**	-7.79	**0.01**	-8.97	**<0.001**	-7.24	**0.05**
	(-14.64/-1.74)		(-15.37/-2.22)		(-15.57/-2.37)		(-14.62/0.13)	
**Left Ankle Plantar flexion**								
adjusted r ^2^	0.0103		-0.0071		-0.0021		0.0487	
HTLV-1 ^(+)^	-5.47	0.12	-6.07	0.10	-5.82	0.11	0.97	0.83
	(-12.57/1.62)		(-13.37/1.22)		(-13.12/1.47)		(-8.17/10.1)	
TSP/HAM	-4.23	0.14	-4.29	0.14	-4.48	0.12	-3.25	0.39
	(-9.91/1.42)		(-10.12/1.54)		(-10.31/1.35)		(-10.85/4.36)	

**Abbreviations:** 95% CI, 95% confidence interval; HTLV-1, human T cell lymphotropic virus type 1; TSP/HAM, tropical spastic paraparesis / HTLV-1-associated myelopathy; IPAQ, international physical activity questionnaire. **Notes:** Model 1: adjusted for age; Model 2: adjusted for age, sex, and body mass index; Model 3: adjusted for age, sex, BMI, and physical activity level; Model 4: adjusted for age, sex, BMI, IPAQ, and low back pain. All comparisons were performed using the Kruskal-Wallis test and Dunn-Bonferroni post-test correction.

A subcomparison of the differences in knee and ankle movement between the TSP/HAM and HTLV-1 ^(+)^ groups revealed significantly worse values in all models with respect to left knee flexion movement (data not shown).

## DISCUSSION

Flexibility is significantly reduced in individuals with TSP/HAM as assessed by the pendular fleximeter. Significantly lower flexibility was noted in the trunk extension and flexion, and bilateral hip extension movements compared with individuals without myelopathy and HTLV-1 ^(−)^ subjects. After adjusting for confounding variables, such as age, sex, BMI, level of physical activity, and lower back pain, multivariate analysis confirmed that individuals with TSP/HAM presented significantly reduced flexibility in approximately 50% of the joint movements studied, when compared with the HTLV-1 ^(−)^ reference group. Some causal factors underlying the reduction in flexibility observed in these patients could include neurological disorders associated with TSP/HAM, such as spasticity, muscle shortening, and weakness, postural changes, and reduced mobility. 16 38 39 40

In patients with TSP/HAM, decreased mobility was observed in the hip joint in terms of flexion, extension, external rotation, and abduction movements, when compared with the HTLV-1 ^(-)^ group. These types of motor alterations may be explained by the clinical picture of TSP/HAM, as it is already known that the region proximal to the lower limb muscles is mainly affected in patients with spastic paraparesis, generally characterized by muscle shortening and stiffness. 36 While the present study did not specifically investigate these symptoms in the present sample, it would be advisable for future studies to investigate the potential relationship in HTLV-1 ^(+)^ individuals with TSP/HAM in a prospective cohort.

TSP/HAM patients present pelvic instability due to muscle shortening and the inability to resist gravity due to muscle weakness. 16 41 This occurs due to changes in the lumbopelvic musculature, such as the paravertebral, core muscles, and the muscles of the pelvis, such as the hamstrings, glutes, and iliopsoas, which may lead to an inability to resist gravity and maintain a standing posture. 16 41 Furthermore, pelvic instability is characterized by an inability of the passive and, especially, the active systems to control movements in the region, and to maintain the balance and firmness necessary for lumbopelvic movements to occur without overload. 42 Caiafa et al (2016) stated that muscle weakness impacted lower limb movement more than spasticity. 39 They observed weakness in the flexor, abductor, and adductor muscles of the hip and knee flexors, which led to loss of pelvic control and gait imbalance in individuals with TSP/HAM. 39 Biomechanical compensations resulting in the misalignment of the lower limbs are known to occur when the pelvis loses the ability to remain in a neutral position. 43

Pelvic instability can also lead to a loss of knee flexion in patients with TSP/HAM, as biomechanical compensatory mechanisms change the center of the knee. 16 The postural pattern of individuals with TSP/HAM shifts the body forward, leading to anteriorization of the head and trunk, reduced hip and knee flexion, as well as ankle angulation. 16 39 Since the plantar flexor muscles are affected by spasticity in patients with TSP/ HAM, 16 39 it is possible to infer that the observed repercussions in dorsiflexion flexibility may also be affected in these individuals.

Among patients living with HTLV-1 who did not demonstrate clinical signs of myelopathy presented decreased plantar flexion compared with uninfected individuals after adjusting for sex, age, and physical activity. This finding suggests that some degree of impairment in plantar flexion may already be occurring in patients living with HTLV-1, despite the absence of other myelopathic signs or symptoms. Indeed, previous reports have shown clinical manifestations occurring in HTLV-1-infected patients without myelopathy, and it has been postulated that even asymptomatic HTLV-1 carriers may harbor other neurological signs and symptoms that potentially constitute early indicators of myelopathy development, such as subcortical cognitive deficits. 13 32 44

Lower back pain is the most frequently reported pain in patients with HTLV-1. 45 This may be related to muscle impairment (spasticity and muscle shortening), postural deviation, and joint immobility, 45 which can result in locomotor disability, decreased quality of life and a lower level of functional activity. 38 46 47 However, the present study did not identify lower back pain as an independent factor associated with flexibility.

In contrast to a study conducted by Santos, 2015, 20 we identified no differences in flexibility between groups with regard to the sit-and-reach test. These authors found that noninfected individuals presented superior performance in the sit-and-reach test compared with the TSP/HAM group, while the opposite was true in our study: the HTLV-1 ^(-)^ group presented inferior results compared with the TSP/HAM group. It is possible that the TSP/HAM group exhibited superior performance as a result of the regular physical therapy (supervised physical therapy once a week, and at-home exercises from the HTLV-1 exercise book guide) provided to these patients. 48 A limitation of this study was the lower number of individuals in the HTLV-1 ^(+)^ without myelopathy and HTLV-1 ^(-)^ groups. Another limitation is the lack of associations with specific factors that underline the loss of flexibility in individuals with myelopathy. Nonetheless, it is important to emphasize that some individuals in the HTLV-1 ^(+)^ group without myelopathy presented a loss of flexibility despite not being classified as TSP/HAM, which may indicate that muscle weakness can more relevantly impact flexibility than spasticity. This warrants further investigation. Finally, while muscle weakness was not investigated herein, we recommend that future studies include this parameter in an attempt to elucidate factors underlying the observed loss in flexibility in patients living with HTLV-1.

Because the number of recruits in these groups was lower than the estimated calculation, the power of the study was reduced by increasing the β error. Therefore, the hypothesis that asymptomatic subjects suffering from myelopathy have limited flexibility cannot be excluded. However, as mentioned above, decreased flexibility was observed in certain joints. New studies should be performed to confirm these differences.

It is important to note that no broad consensus exists regarding the most appropriate method of measuring flexibility. However, the pendular fleximeter has been suggested as a more accurate tool to assess flexibility, when compared with the sit-and-reach test. Thus, it is necessary to comprehensively assess the accuracy of instruments designed to assess flexibility.

It can be concluded that individuals with TSP/HAM present reduced joint flexibility in most movements evaluated by the pendulum fleximeter. In addition, patients infected with HTLV-1 subjects without myelopathy demonstrated loss of flexibility in some joints, such as the knee and ankle. In view of these results, it is highly recommended that careful evaluations of flexibility to be longitudinal in patients with HTLV-1, to better understand the impacts of myelopathy in reduced mobility and, consequently, in the quality of lives of people living with HTLV-1.
